# Characterization of the release and biological significance of cell-free DNA from breast cancer cell lines

**DOI:** 10.18632/oncotarget.17858

**Published:** 2017-05-15

**Authors:** Wei Wang, Peng Kong, Ge Ma, Li Li, Jin Zhu, Tiansong Xia, Hui Xie, Wenbin Zhou, Shui Wang

**Affiliations:** ^1^ Department of Breast Surgery, The First Affiliated Hospital with Nanjing Medical University, Nanjing 210029, China

**Keywords:** breast cancer, cell-free DNA, circulating tumor DNA, biological significance

## Abstract

In breast cancer, cell-free DNA (cfDNA) has been proven to be a diagnostic and prognostic biomarker. However, there have been few studies on the origin and biological significance of cfDNA. In this study, we assessed the release pattern of cfDNA from breast cancer cell lines under different culture conditions and investigated the biological significance of cfDNA. The cfDNA concentration increased rapidly (6 h) after passage, decreased gradually, and was then maintained at a relatively stable level after 24 h. In addition, the cfDNA concentration did not correlate with the amount of apoptotic and necrotic cells. Interestingly, if more cells were in the G1 phase, more cfDNA was detected (p < 0.01) and the cfDNA concentration correlated positively with the percent of cells in the G1 phase (p < 0.05). We observed that cells could release cfDNA actively, but not exclusively, via exosomes. Furthermore, we showed that cfDNA could stimulate hormone receptor-positive breast cancer cell proliferation by activating the TLR9-NF-κB-cyclin D1 pathway. In conclusion, cfDNA is released from breast cancer mainly by active secretion, and cfDNA could stimulate proliferation of breast cancer cells.

## INTRODUCTION

DNA fragments in circulation released by cells are referred to as cell-free DNA (cfDNA), and cfDNA carrying tumor specific sequence alterations is known as circulating tumor DNA (ctDNA) [1]. CfDNA, especially ctDNA, has been acknowledged as a potential diagnostic and prognostic biomarker for several types of cancer [2–4]. Although cfDNA is ubiquitous, its generative mechanism remains incompletely determined [5, 6].

Several studies suggest an apoptotic origin of cfDNA from cancer cells in patients with an electrophoretic ladder pattern and fragment sizes from 150 to 1000 bp [7, 8]. Interestingly, other researchers have observed longer DNA fragments (> 1000 bp) in the blood of colorectal cancer patients compared with healthy cases [6]. They speculated that cfDNA originates from necrotic cells, because necrotic cells could release more long DNA fragments into circulation [9]. However, another study showed that patients receiving radiation therapy, which induces mainly cell necrosis, had a 90% reduction in cfDNA levels, arguing against necrosis as the primary pathway for cfDNA release [10]. Additionally, several studies have indicated that cfDNA is derived from active cellular secretions, such as exosomes, apoptotic blebs, shedding vesicles, and microparticles [11, 12]. Furthermore, many *in vivo* confounding factors, which are circumvented partially by *in vitro* models [13], may affect the release of cfDNA. Therefore, apoptosis, necrosis, and active cellular secretion seem to partly account for the occurrence of cfDNA; however, the exact mechanism of cfDNA release remains elusive, especially in breast cancer.

Importantly, some studies identified biological effects of circulating cell-free nucleic acids. For example, microRNAs in blood have important functions in tumorigenesis, metastasis and resistance [14]. However, there are very few reports on the effects of cfDNA on cancer cells. Garcia-Olmo et al. reported that cfDNA from colon adenocarcinoma cells could promote tumor metastasis and proposed the “genometastasis” hypothesis [15]. In subsequent studies, researchers found that cfDNA from colorectal tumor patients could induce oncogenic transformation of NIH-3T3 cells and adipose-derived stem cells [16]. In breast cancer, few studies have investigated the biolo gical significance of cfDNA. Tuomela et al. showed that DNA from dead cancer cells could induce invasion and inflammation of breast cancer cells [17].

In the present study, to avoid *in vivo* confounding factors, we assessed the released pattern of cfDNA from cultured human breast cancer cells under different culture conditions and identified the critical factors that influence cfDNA release *in vitro*. Furthermore, we observed that cfDNA could promote the proliferation of hormone receptor positive (HR+) breast cancer cells by the activating toll-like receptor 9 (TLR9)-nuclear factor kappa B (NF-κB)-cyclin D1 pathway.

## RESULTS

### Kinetics of cfDNA release under normal culture conditions

**Figure 1:**
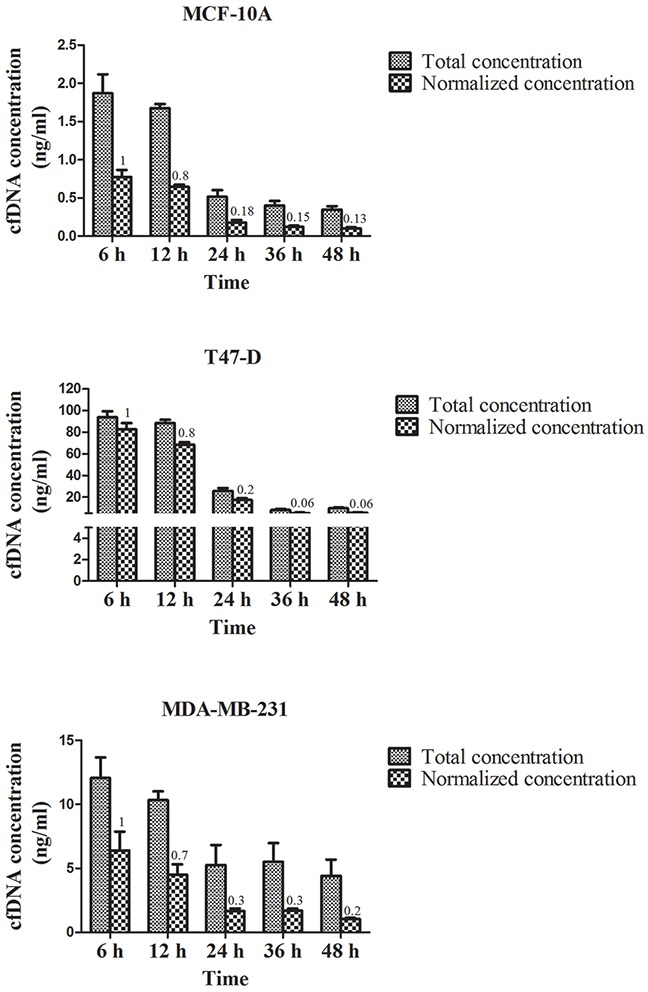
CfDNA concentration in the supernatant of MCF-10A, T47-D, and MDA-MB-231 cell lines under normal culture conditions The number at the top of each bar represents the relative concentration at each time point. Total concentration: the cfDNA concentration in the supernatant of breast cancer cells. Normalized concentration: the cfDNA concentration was normalized in terms of the amount of viable cells.

We found the cfDNA concentration from breast cancer cell lines (T47-D and MDA-MB-231) was higher than that from a normal mammary gland cell line (MCF-10A) under regular culture conditions at all time points. Besides, the amount of cfDNA released by T47-D cells was higher than that released by MDA-MB-231 cells, and this tendency did not change when the DNA concentration was normalized in terms of the amount of total viable cells. After growth medium renewal, the total amount or the normalized concentration of cfDNA released by T47-D and MDA-MB-231 cells increased incrementally after 6 h, decreased gradually over time, and then plateaued after 24 h (Figure 1).

### The cfDNA concentration had no correlation with cellular apoptosis and necrosis

**Figure 2:**
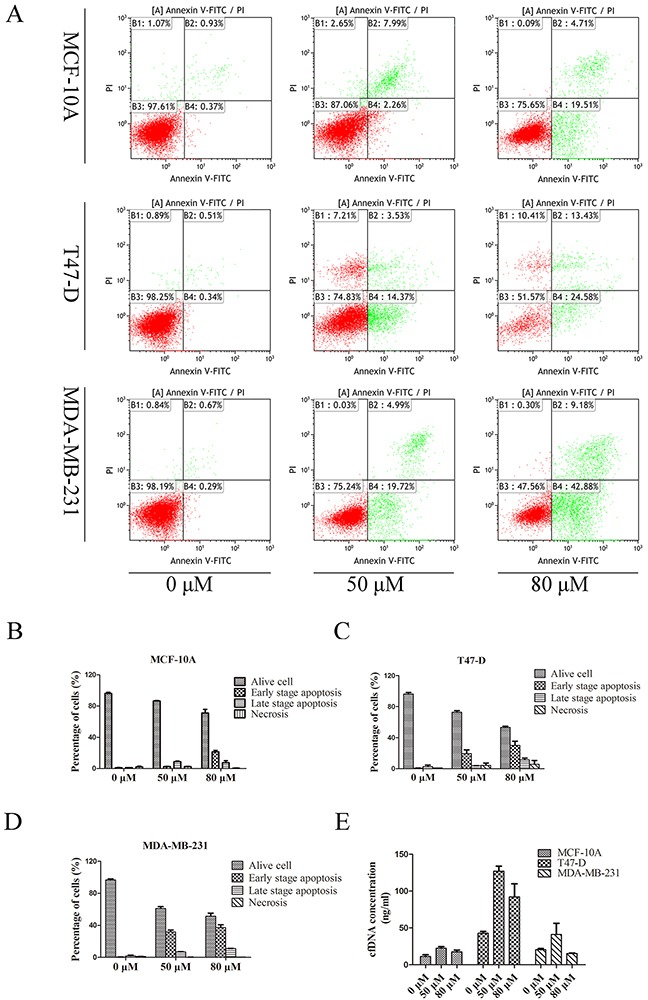
Concentration of cfDNA from breast cancer cells with different levels of apoptosis and necrosis **(A)** Scatter diagram of the flow cytometry of cells treated with different doses of the apoptotic inducer; **(B–D)** histograms of the apoptosis and necrosis ratio of MCF-10A, T47-D, and MDA-MB-231 cells; **(E)** CfDNA concentration of cell lines treated with different doses of the apoptotic inducer.

To investigate the relationship between the cfDNA concentration and cellular apoptosis or necrosis, T47-D, MDA-MB-231, and MCF-10A cells were treated with 0, 50, 80 μM cisplatin, respectively, to induce different levels of apoptosis and necrosis. Flow cytometry showed that the corresponding mean apoptotic rate of MCF-10A cells under the three concentrations of cisplatin was 1.80, 11.17, and 28.65%; for T47-D cells it was 3.00, 22.92, and 41.26%; and for MDA-MB-231 cells it was 2.22, 38.79, and 48.19%. The mean necrotic rate of MCF-10A cells under the three concentrations of cisplatin was 1.95, 2.36, and 0.37%; for T47-D cells it was 0.85, 4.25, and 5.57%; and for MDA-MB-231 cells it was 1.04, 0.17, and 0.33%. Concentration analysis showed the corresponding cfDNA concentrations released by MCF-10A cells were 11.36, 22.38, and 17.55 ng/mL; for T47-D cells they were 42.91, 127.07, and 92.18 ng/mL; and for MD-MB-231 cells they were 20.42, 41.16, and 15.39 ng/mL (Figure 2). Correlation analysis suggested that cfDNA concentration had no definitive correlation with the extent of cellular apoptosis (correlation coefficients: MCF-10A: 0.409, p = 0.731; T47D: 0.602, p = 0.589; MDA-MB-231: 0.143, p = 0.909) or necrosis (correlation coefficients: MCF-10A: 0.125, p = 0.920; T47D: 0.765, p = 0.445; MDA-MB-231: 0.491, p = 0.674).

### Cells in G1 phase had a positive correlation with cfDNA concentration

**Figure 3:**
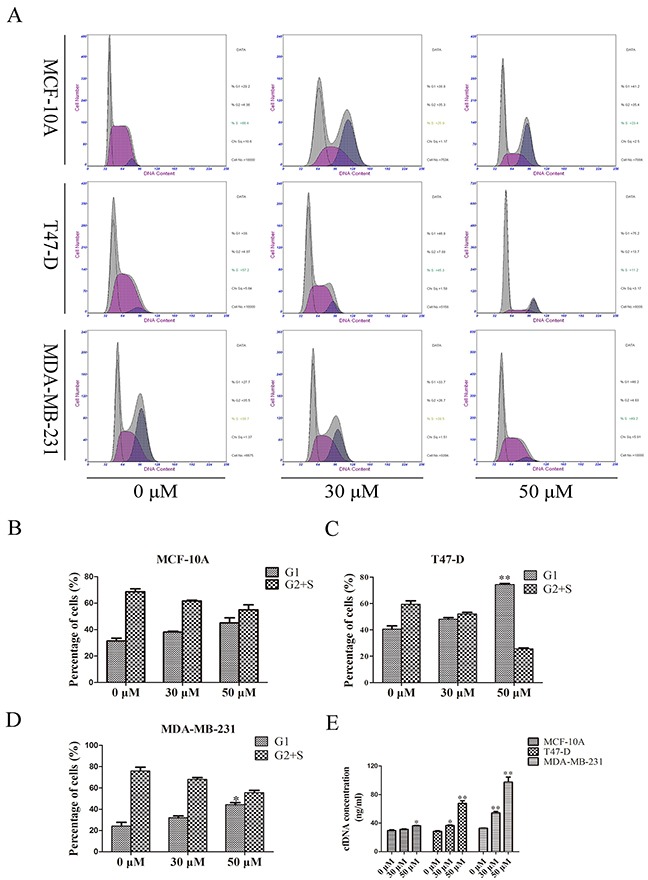
Relationship of cfDNA concentration and cell-cycle **(A–D)** Cell-cycle analysis of three cell lines treated with different doses of cell-cycle inhibitor; **(E)** CfDNA concentration of cell lines treated with different doses of cell-cycle inhibitor.

To investigate whether the origin of cfDNA was influenced by cell cycle, MCF-10A, T47-D, and MDA-MB-231 cells were treated with 0, 30, and 50μM roscovitine. Cell-cycle analysis showed that under these three roscovitine concentrations, 31.35, 38.2, and 45.05% of MCF-10A cells; 40.5, 48.05, and 74.45% of T47-D cells; and 24.1, 31.95, and 44.25% of MDA-MB-231 cells stayed in the G1 phase, respectively. Concentration analysis showed the corresponding cfDNA concentrations of MCF-10A cells were 29.52, 31.38, and 36.24 ng/mL, from T47-D cells were 28.58, 36.79, and 67.63 ng/mL; and from MDA-MB-231 cells were 32.96, 54.43, and 97.46 ng/mL (Figure 3). Correlation analysis suggested the percent of cells in G1 phase correlated positively with the cfDNA concentration (correlation coefficients: MCF-10A: 0.968, p = 0.161; T47D: 1.00, p = 0.008; MDA-MB-231: 0.998, p = 0.041). To confirm the relationship between the proportion of cells in the G1 phase and the cfDNA concentration, we also cultured cells in Dulbecco's modified Eagle medium (DMEM) with 10, 2.5, and 0% fetal bovine serum (FBS) to induce cell-cycle changes. The results confirmed that the percent of cells in G1 phase correlated positively with the cfDNA concentration (Supplementary Figure 2).

### Cells in the G1 phase released cfDNA mainly, but not exclusively, through exosomes

**Figure 4:**
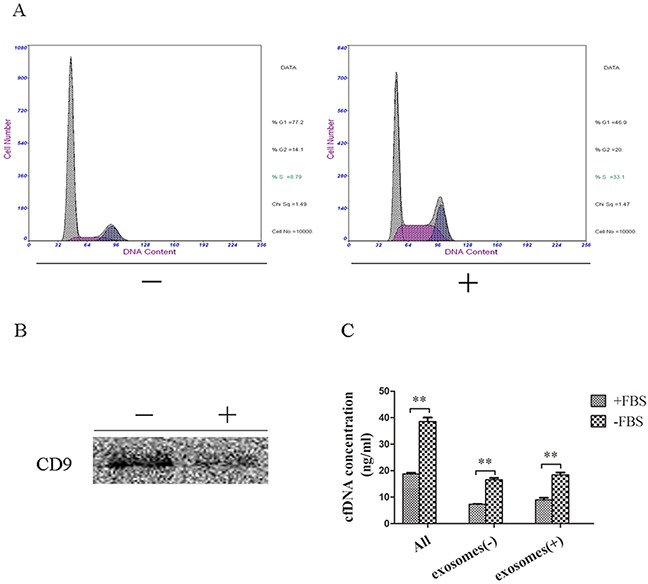
CfDNA concentration of exosomes and the corresponding culture supernatant **(A)** Cell-cycle analysis of cells cultured with or without FBS; **(B)** CD9 expression of exosomes extracted from the supernatant with or without FBS; **(C)** CfDNA concentration of total supernatant (all), exosomes (exosomes (+)) and supernatant without exosomes (exosomes (−)). +: culture with FBS; —: culture without FBS.

The cfDNA concentration was not influenced by the amount of apoptotic or necrotic cells, but was associated with the amount of viable cells. Therefore, we next assessed the relationship between cfDNA concentration and exosomes, an active secretion from viable cells, to explore whether cells released cfDNA mainly through exosomes. When cultured without FBS, more cells stayed in the G1 phase. In addition, the protein level of CD9 (a marker of exosomes) in the culture supernatant without FBS was higher than in the supernatant with FBS (Figure 4A, 4B). Concentration analysis showed that the total concentration of cfDNA in the supernatant without FBS (39.37 ng/mL) was significantly higher than that in the supernatant with FBS (18.94 ng/mL) (p < 0.01). The cfDNA concentration associated with exosomes in the supernatant without FBS was significantly higher than that from supernatant with FBS (18.39 ng/mL *vs*. 8.94 ng/mL, p < 0.01). Similarly, in the remaining supernatant after eliminating exosomes, the cfDNA concentration in the FBS negative group remained significantly higher than that in the FBS positive group (16.61 ng/mL *vs*. 7.27 ng/mL, p < 0.01). However, the difference in the cfDNA concentration between exosomes and corresponding supernatant without exosomes was not statistically significant (p > 0.05), in both the FBS negative group (18.39 ng/mL *vs*. 16.61 ng/ml, p = 0.19) and the FBS positive group (8.94 ng/mL *vs*. 7.27 ng/mL, p = 0.11) (Figure 4C). Thus, the results suggested that cells in the G1 phase could release cfDNA via exosomes, but not exclusively.

### CfDNA promoted the proliferation of HR+ breast cancer cells

**Figure 5:**
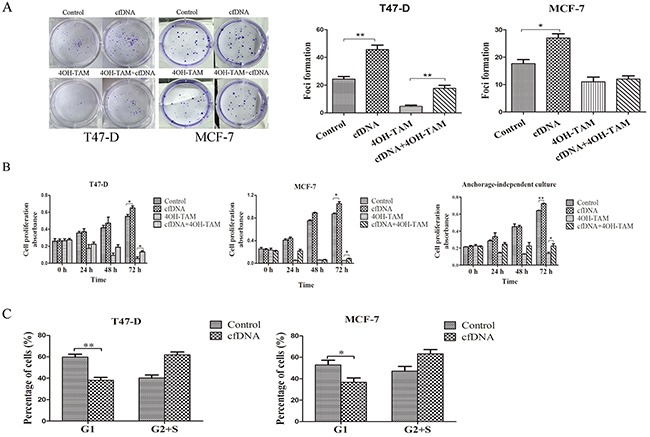
The promotion of T47-D and MCF-7 cells proliferation by cfDNA **(A)** Clone formation assay of cells treated with cfDNA; **(B)** CCK-8 assay of cells treated with cfDNA; **(C)** cell-cycle analysis of cells treated with cfDNA.

To explore the biological significance of cfDNA in breast cancer cells, T47-D and MDA-MB-231 were treated with cfDNA extracted from the supernatant of MD-MB-231 breast cancer cells. Clone formation assays and CCK-8 proliferation assays showed that cfDNA could not promote the proliferation of MDA-MB-231 cells (Supplementary Figure 3). In contrast, the clone formation assay showed that the number of foci formed by T47-D cells increased significantly after cfDNA was added into medium compared with the control group (mean foci number: 46 *vs*. 24, p < 0.01). When T47-D cells were treated with 4-hydroxy-tamoxifen (4OH-TAM), the number of foci decreased significantly; however, the cfDNA plus 4OH-TAM group had more foci than the 4OH-TAM group (18 *vs*. 5, p < 0.05) (Figure 5A). The results of the CCK-8 proliferation assay were consistent with those of the clone formation assay. In addition, the proliferative ability of T47-D cells in anchorage-independent culture conditions also was assessed and the results were similar to those in anchorage-dependent conditions (Figure 5B). Cell-cycle analysis showed that the number of T47-D cells in the G1 phase declined significantly when they were incubated with cfDNA (p < 0.01) (Figure 5C). Similarly, we found that cfDNA could promote the proliferation of MCF-7 cells (p < 0.05) (Figure 5).

### CfDNA promoted HR+ breast cancer cell proliferation due to activate the TLR9-NF-κB pathway

**Figure 6:**
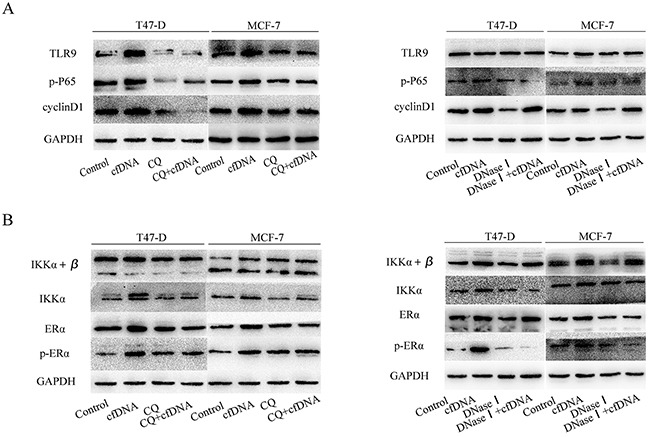
Protein expression levels of TLR9-NF-κB pathway members after the cells were treated with cfDNA **(A)** Protein expression levels of TLR9, p-P65, and cyclin D1 in T47-D and MCF-7 cells after treatment with cfDNA, CQ, cfDNA+CQ, DNase I, DNaseI+cfDNA; **(B)** protein expression levels of IKK α + β, IKK α, ERα, and p- ERα in T47-D and MCF-7 cells after treated with cfDNA, CQ, cfDNA+CQ, DNase I, DNase I+cfDNA. CQ: chloroquine; p-P65: phosphorylated P65; p-ERα; phosphorylated Erα.

We next explored the underlying molecular mechanism by which cfDNA promotes the proliferation of HR+ breast cancer cells. Endogenous DNA is recognized by TLR9 [18], and the TLR9-NF-κB pathway has an important function in cancer cell proliferation. Therefore, we assessed the TLR9-NF-κB pathway and found that cfDNA could activate the TLR9-P65 pathway, ultimately increased the protein expression of cyclin D1. To prove that cfDNA was definitively recognized by TLR9, chloroquine, a TLR9 inhibitor, was used to block the binding of the two molecules. The results showed that the levels of phosphorylated P65 (p-P65) and cyclin D1 decreased after TLR9 inhibition compared to the control and cfDNA could not increased the protein expression of downstream molecules. In addition, we found that Dnase I, which can digest DNA sufficiently (Supplementary Figure 4), could inhibit the cfDNA-dependent increase in protein expression (Figure 6A). Collectively, we showed that cfDNA could promote the proliferation of HR+ breast cancer cells via the TLR9-NF-κB-cyclin D1 pathway.

Furthermore, we found that the protein levels of estrogen receptor alpha (ERα), another upstream molecule of cyclin D1, were increased in T47-D and MCF-7 cells treated with cfDNA. Although several studies have reported that TLR9 correlates negatively with ERα [19], others reported the opposite [20]. Park et al. have reported that an IκB kinase α (IKKα)-dependent transcription complex was required for ERα-mediated gene activation [21]. Therefore, we evaluated the protein expression levels of IKKα + β, IKKα, ERα, and phosphorylated ERα (p-ERα), and found they were increased after treatment with cfDNA (Figure 6B). Thus, we showed that cfDNA could promote HR+ breast cancer cell proliferation by activating the TLR9-NF-κB pathway directly and indirectly. Additionally, inflammatory response in the cfDNA group was not remarkable compared with the control group in this study (Supplementary Figure 5).

## DISCUSSION

In breast cancer, cfDNA, or ctDNA, has been demonstrated to be a promising diagnostic and prognostic biomarker [3, 22, 23]. However, most studies concerned its diagnostic capability for cancer and paid little attention to its release pattern [4, 24, 25]. Although previous studies have reported that the occurrence of cfDNA is associated with apoptosis or necrosis *in vivo*, these might be influenced by the complex internal environment [13].

In the present study, we first assessed the release pattern of cfDNA from human breast cancer cells using an *in vitro* model to eliminate confounding factors. In addition, we also investigated whether cfDNA has a direct biological influence on cancer cells. We found that the cfDNA concentration increased in a short time after passage, decreased gradually, and was then maintained at a relatively stable level in normal culture conditions. Besides, T47-D cells, considered to be less malignant breast cancer cells [26, 27], released more cfDNA than MDA-MB-231 cells. When cells were treated with different doses of an apoptosis inducer, the cfDNA concentration did not correlate with the amount of apoptotic and necrotic cells. In contrast, correlation analysis suggested the percent of cells in G1 phase correlated positively with the cfDNA concentration. We also found that cells in the G1 phase could release cfDNA through exosomes. However, this accounted for only a part of the total released cfDNA. Furthermore, we showed cfDNA could promote HR+ breast cancer cell proliferation by activating the TLR9-NF-κB-cyclin D1 pathway.

Until now, the mechanism of cfDNA release was unclear [28]. Several studies showed that cfDNA was released mainly by necrotic cancer cells and comprised more long DNA fragments compared with that from normal cells. Necrosis is a common event in tumor environment and necrotic cells could release more undigested, longer DNA fragment into circulation. However, other reports supported the view that cfDNA is released mainly from apoptotic tumor cells, because they found the shorter DNA molecules in blood that carried tumor-associated copy number aberrations preferentially [6, 8]. Although there seems to be more evidence to support the apoptotic theory, the exact mechanism remains inconclusive. Under physiological conditions, most cfDNA would be degraded by DNase I in the blood. Only when the balance of generation and degradation is altered would more cfDNA be detected [29]. This explains why the cfDNA concentration declined gradually, and was then maintained at a relatively low level under regular cultured condition, whereas it increased when cells were treated with low dose of an apoptotic inducer in this study. However, we showed that as more apoptotic and necrotic cells appeared, the cfDNA concentration declined. This seemed to contradict what we expected. We speculated that when a large amount of cell lysis occurs, DNase (DNase II, DNase III) inside the cells would also be released into the blood, where it could digest the increased cfDNA [30]. For the undigested cfDNA, cells might have some protective mechanism. Some studies have shown that cancer cells actively secrete cfDNA into the blood circulation in different forms, such as in exosomes, and the cfDNA inside these carriers could be protected from degradation [12, 14]. Our results also suggested that breast cancer cells in G1 phase secrete more exosomes. However, the cfDNA inside the exosomes only accounted for a part of total cfDNA, suggesting the presence of other protective mechanisms. Our observations partially proved that the increase in cfDNA was not associated with the process of DNA replication, but was the consequence of active release from differentiated cells, because differentiated cells tend to be held in the G1 phase [31].

Several studies have confirmed that cfDNA is released from cells to act as an intercellular messenger. When cfDNA enters target cells, it is either integrated into the host genome or bind to receptors to elicit a biological effect, such as induction of tolerance against detrimental substances, immunomodulation, development of metastasis, and generation of genetic instability [32, 33]. In breast cancer, Tuomela et al. found DNA from dead cancer cells could induce TLR9-mediated invasion and inflammation in living MDA-MB-231 cells [17]. However, that study only reported an observed phenomenon in a triple negative breast cancer cell line and did not further explore the underlying mechanism. TLR9 is an innate immune system effector and a cellular DNA-receptor that can not only recognize microbial or viral DNA, but also endogenous DNA. Stimulation of TLR9 can induce an NF-κB-mediated inflammatory response that has a critical role in autoimmune disease and cancers [34]. In this study, we found that cfDNA from breast cancer cells not only could activate the TLR9-NF-κB pathway, but also increased the amount of p-ERα in HR+ breast cancer cells, ultimately increasing the abundance of cyclin D1. However, we did not observe a significant increase of inflammatory cytokine levels after the cells were treated with cfDNA. This might reflect the fact that an inflammatory response is not a common event in HR+ breast cancer [35].

Cyclin D1 is an effect molecular of NF-κB or ERα. It can be activated by NF-κB and ERα, respectively; however, the interaction between NF-κB and ERα remains controversial. Although the majority of studies suggested a reciprocal inhibition between ERα and NF-кB [36], some studies have demonstrated positive cross-talk between these transcription factors [37]. The inconsistency remains to be resolved. Frasor et al. suggested that a synergistic interaction and a trans-repressive interaction could occur together; however, the positive cross-talk was more extensive in breast cancer cells [20]. NF-κB proteins are often located in the cytoplasm, where they are bound to the inhibitory protein, IκB. When cells are exposed to a variety of extracellular stimuli, such as endogenous DNA, the IκB kinase (IKK) complex (including KKα and IKKβ) can be activated, resulting in the degradation of IκB proteins. This process leads to the translocation of NF-κB proteins from the cytoplasm to the nucleus, where these proteins can activate the expression of NF-κB-regulated genes [38]. Park et al. showed that activated IKKα could also lead to the enhanced phosphorylation of ERα. IKKα and ERα were recruited to the promoter region of ERE-regulated genes, resulting in the activation of estrogen-mediated transcription [21].

The present study also had some limitations. First, the *in vitro* model could not completely represent the actual condition of patients; therefore, our findings only provided limited information concerning the biogenesis of cfDNA and further in depth studies are needed. Second, the effect of cfDNA on cell proliferation was investigated only in HR+ breast cancer cell lines. We hypothesized that there might be other influencing factors and more studies should be performed on the effect of cfDNA on other molecular subtypes of breast cancer.

In conclusion, we found the cfDNA was released by breast cancer cells mainly via active cellular secretion. In addition, cfDNA could stimulate the proliferation of HR+ breast cancer cells by activating the TLR9-NF-κB-cyclin D1 pathway.

## MATERIALS AND METHODS

### Cell lines and cell culture

Human T47-D, MDA-MB-231, and MCF-10A cells were purchased from the American Type Culture Collection (ATCC, USA). MCF-10A is a normal breast epithelial cell line. T47-D is a estrogen receptor (ER) positive, progesterone receptor (PR) positive or negative, human epidermal growth factor receptor 2 (HER2) negative breast cancer cell line and MDA-MB-231 is a ER negative, PR negative, HER2 negative breast cancer cell line [26]. Cells were cultured in DMEM (Life Technologies, UK) supplemented with 10% heat-inactivated FBS (Gibco, USA) and 1% penicillin/streptomycin (GE Healthcare Life Sciences, USA). All cells were cultured in humidified atmosphere containing 5% CO_2_ at 37°C. After 1–2 generations, cells were detached from plates and then used for subsequent experiments.

### Cell culture under normal conditions

Cells (10^5^ cells per well) were plated into 12-well plates and cultured for 24 h. Thereafter, the growth medium was discarded. Cells were washed twice with sterile phosphate buffered saline (PBS) and incubated in 2 mL of fresh medium for 6, 12, 24, 36, and 48 h respectively.

### Induction of apoptosis, necrosis, and cell-cycle changes

Cells were plated into 6-well plates and cultured to 80% confluence. The cells were then washed twice with PBS and incubated for 24 h in 2 mL fresh medium with different doses of apoptosis inducer (cisplatin, 0, 50, and 80 μM) or cell-cycle inhibitor (roscovitine, 0, 30, and 50 μM) (Beyotime Biotechnology, China).

At the end of the incubation, the growth medium was collected in 2 mL nuclease-free tubes (Eppendorf, Germany) and centrifuged at 2000 × g for 20 min and 1 mL of supernatant was transferred to a fresh 1.5 mL tube. The samples were then stored at −80°C until use. Cells were detached from the plates and then used for subsequent analyses.

### Flow cytometry

#### Measurement of apoptosis and necrosis

Cells were washed twice with PBS and re-suspended in 1 × binding buffer at a concentration of 1 × 10^6^ cells/mL. Then, 200 μL of this solution were transferred to round bottom tubes and mixed with 3 μL of fluorescein isothiocyante (FITC) Annexin V and 3 μL of propidium iodide (PI) (BD Biosciences, USA). Samples were vortexed and incubated in the dark at room temperature for 20 min. After incubation, cells were analyzed using a flow cytometer (BD Biosciences, USA).

#### Measurement of cell-cycle

Cell-cycle analysis was done according to the protocol of a commercial kit (MultiSciences, China). The cells (10^6^) were washed twice with PBS and re-suspended in 1 mL of room temperature PBS. Then, 100% ethanol (3 mL) at −20°C was mixed with the cell suspension, which was stored at −20°C until use. Before cell-cycle analysis, the cells were centrifuged at 1000 × rpm for 3 min and the supernatant was discarded. The pelleted cells were hydrated in 2 mL of room temperature PBS for 15 min. DNA staining solution (1mL) was added into the collection tube and mixed by vortexing for 10 s. The sample was then incubated in the dark for 30 min before analysis using the flow cytometer.

#### Extraction of exosomes from cultured medium

MDA-MB-231 cells were cultured to 80% confluence, then washed twice with PBS and incubated for 48 h in fresh medium with or without FBS. Then, cell-cycle analysis was performed and the supernatant was used to extract exosomes according to the protocol (GENESEED, China): a. The supernatant was centrifuged at 2000 × *g* at 4°C for 20 min to remove debris. b. The supernatant was transferred into a new collection tube and GSTM Exosome Isolation Reagent was added to the sample. c. The sample was mixed and stored at 4°C overnight. d. The supernatant was then centrifuged at 5000 × *g* at 4°C for 30 min. e. The supernatant (1 mL) was transferred into a new 1.5 mL collection tube. f. The sample was centrifuged at 5000 × g at 4°C for 5 min and the pellet, comprising the exosomes, was retained. g. The exosomes were resuspended in 200 μL of PBS. All samples were stored at −80°C.

#### DNA extraction

DNA in the supernatant was extracted from 200 μL of medium using a QIAamp DNA Blood Mini Kit (Qiagen, Germany), according to the manufacturer's protocol. In the exosomes fraction, cfDNA was extracted from the exosomes dissolved in PBS, and also from the supernatant without exosomes and unprocessed supernatant. The final eluate was collected and stored at −20°C

#### Absolute quantitative analysis

The concentration of cfDNA was derived by analyzing ALU repetitive elements. For this element, a 111 bp fragment was measured in triplicate using absolute quantitative analysis. Primers were designed according to a previous report [39]. PCR was performed using a FastStart Universal SYBR Green Master mix kit (Rox, Germany), according to the manufacturer's instructions. The reaction had a final volume of 20 μL, containing 10 μL of 2 × SYBR Green, 0.2 μL of 10 μM PCR forward primer, 0.2 μL of 10 μM PCR reverse primer, 1 μL of DNA template, and 8.6 μL of dH_2_O. The thermal cycling conditions comprised 10 min at 95°C, followed by 40 cycles of 15 s denaturation at 95°C, 60 s annealing at 60°C, and 15 s extension at 72°C. The standard curve of ALU concentration-Ct values was constructed using a known-concentration standard (Supplementary Figure 1). The total cfDNA concentration of a sample was deduced from the concentration of the ALU fragment, which was normalized according to the actual volume.

### Cell function assays

#### Clone formation assay

Cells (10^3^ cells per well) were plated into 6-well plates and cultured in fresh medium with cfDNA (10 ng/mL) or PBS as a control. HR+ breast cancer cells also were treated with 4OH-TAM (10^−5^ M) or cfDNA plus 4OH-TAM. CfDNA was extracted from the supernatant of MDA-MB-231 breast cancer cells treated with roscovitine for 24 h. The cfDNA concentration was measured using a nanodrop 2000 device (Quawell, USA), and the concentration used in this study was 10 ng/mL. Three weeks later, the surviving colonies were fixed, stained with crystal violet, and counted.

#### Cell proliferation assay

Cells (5 × 10^3^ cells per well) were plated into 96-well plates and cultured for 24 h. After 24 h, the cells were washed with sterile PBS and incubated in 100 μL of fresh medium with cfDNA (10 ng/mL) or sterile PBS as a control. HR+ breast cancer cells also were treated with 4OH-TAM (10^−5^ M) or cfDNA plus 4OH-TAM. Cell proliferation was assessed at 0, 24, 48, and 72 h using a Cell Counting Kit-8 (CCK-8 (Dojindo, Japan)), according to the manufacturer's instructions. In addition, T47-D cells were cultured in suspension condition as previous reports [40] and the proliferative capacity of cells (5 × 10^3^ cells per well) in anchorage-independent culture conditions was also assessed.

#### Cell-cycle analysis

Cells were plated into 6-well plates and cultured until 60–80% confluence. Cells were then rinsed with sterile PBS and cultured for further 24 h in fresh culture medium with 10 ng/mL cfDNA or the same volume of PBS as a control. Cell-cycle analysis was then performed as described above.

#### Western blotting analysis and DNase I treatment

Cells were treated with 10 ng/mL cfDNA, 50 nmol/mL chloroquine, cfDNA plus chloroquine, and DMSO as a control for 24 h. The cells were then harvested quickly in lysis buffer and clarified by centrifugation. A BCA protein assay reagent kit (Thermo Scientific, USA) was used to measure the protein concentration. Then, western blotting was done. We also used DNase I to digest DNA to further verify the effect of cfDNA. First, agarose gel electrophoresis (AGE) was performed to prove that the DNA had been digested by DNase I. DNase I was added into the supernatant of T47-D and MDA-MB-231 cell, and then cfDNA was extracted. ALU gene and β-actin genes were amplified by PCR as controls. AGE was used to detect the amount of DNA. After that, cells were treated with 10 ng/mL cfDNA, 30 U/mL DNase I, cfDNA plus DNase I, and DMSO as control for 24 h.

#### Detection of inflammatory cytokines

Inflammatory cytokines in the culture supernatant were detected using an enzyme-linked immunosorbent assay Kit (MULTISCIENCES, China), according to the manufacturer's protocol.

### Statistical analysis

Statistical calculations were carried out using the SPSS statistical software package (Version 20.0, SPSS, Inc.). All experiments in this study were repeated in triplicate, and data are presented as the mean ± SEM. Comparisons between two groups were made using Student's t-test. Correlation coefficients were calculated using Pearson analysis for continuous variables. In all figures, asterisks denote significance levels as follows: *p < 0.05, **p < 0.01.

## SUPPLEMENTARY FIGURES



## Abbreviations

cfDNA: cell-free DNA
ctDNA: circulating tumor DNA
HR+: hormone receptor positive
TLR9: toll-like receptor 9
NF-κB: nuclear factor kappa B
p-ERα: phosphorylated estrogen receptor alpha
