# Supplementary material for: Characterization of the release and biological significance of cell-free DNA from breast cancer cell lines
Source: Oncotarget. 2017 May 15;8(26):43180–91. doi: 10.18632/oncotarget.17858 (PMC5522137; doi:10.18632/oncotarget.17858)
Supplement: Supplementary file 1 [file oncotarget-08-43180-s001.pdf]

# Characterization of the release and biological significance of cell-free DNA from breast cancer cell lines

## SUPPLEMENTARY MATERIALS

ALU (111bp)

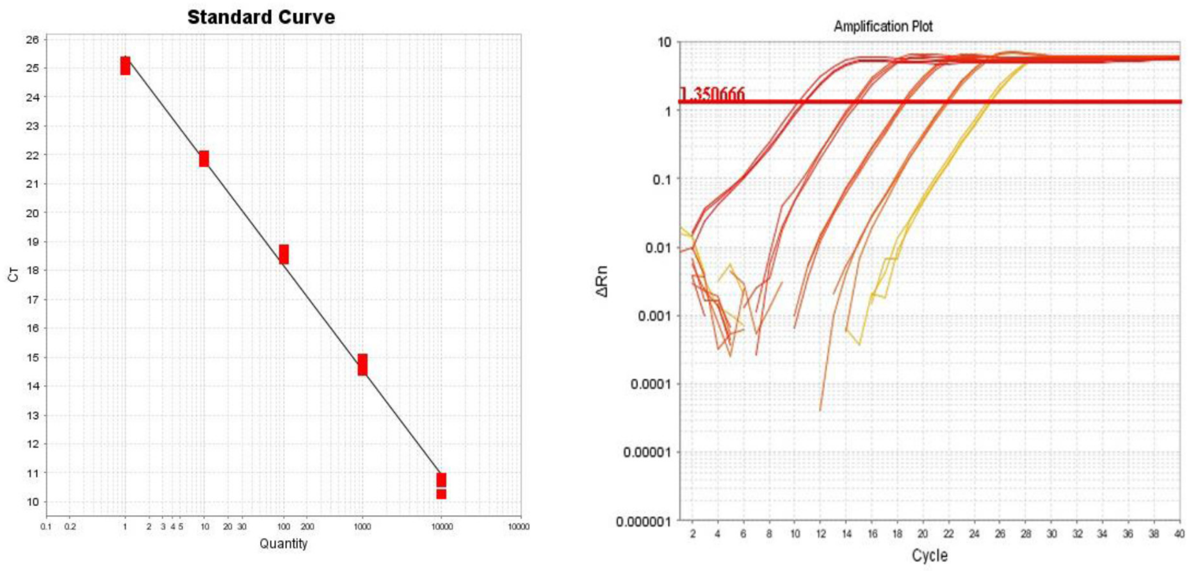


**Supplementary Figure 1: Standard curves of ALU repetitive element (111 bp).** A known concentration of DNA standard was divided to 5 copies and the concentration was diluted to 1 ng/ml, 10 ng/ml, 100 ng/ml, 1000 ng/ml and 10000 ng/ml. Standard curves were done and shown in Figure 1. PCR efficiency was 88.92%. The formula of concentration-Ct value was: Y=-3.62X+25.41, $R^2$=0.996.

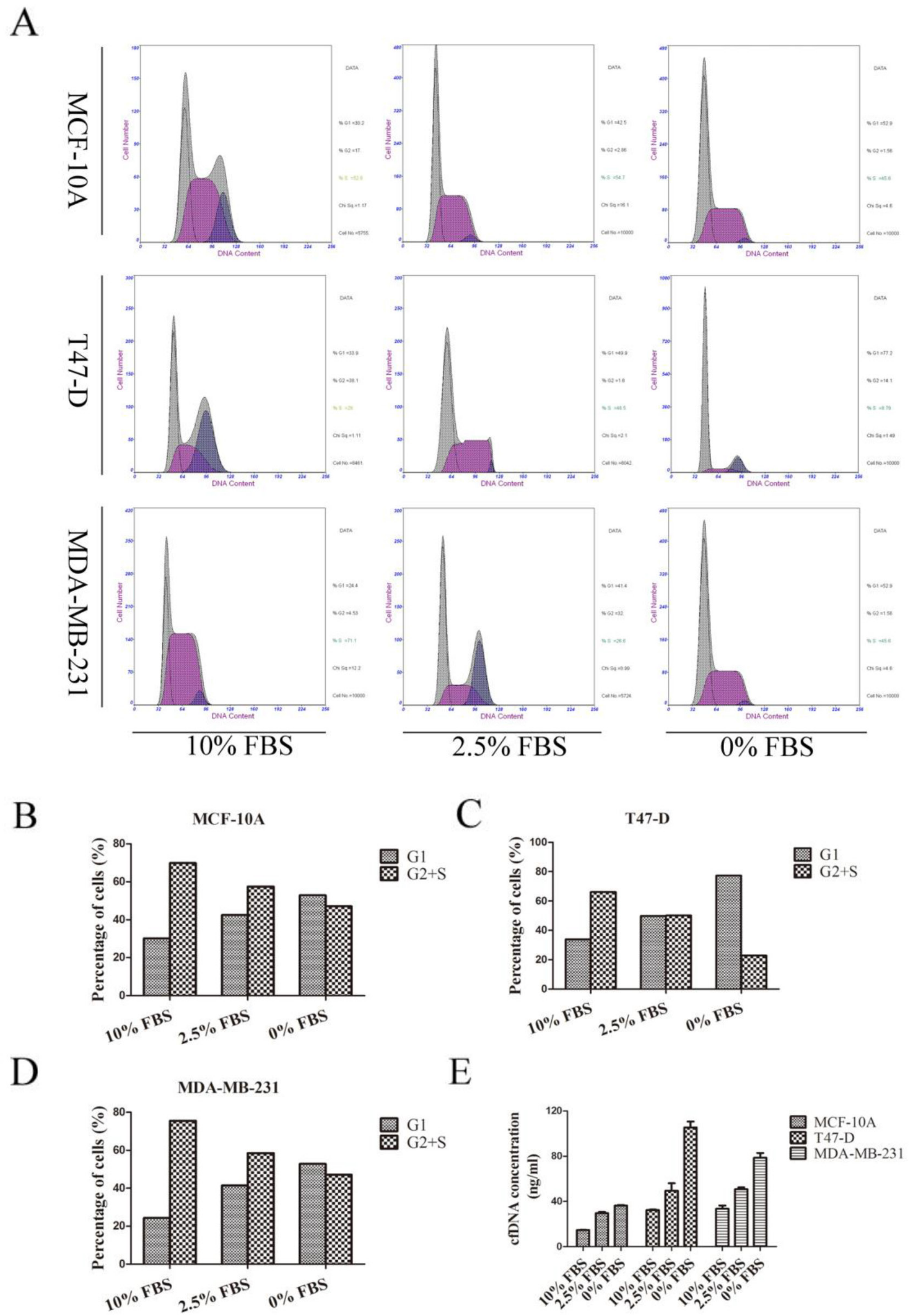


**Supplementary Figure 2: Relationship of cfDNA concentration and cell-cycle.** MCF-10A, T47-D and MDA-MB-231 cells were cultured with 10%, 2.5%, 0% FBS for 48 h. Then cell-cycle and cfDNA concentration were assessed. **(A-D)** Cell-cycle analysis of cells treated with 10%, 2.5%, 0% FBS; **(E)** corresponding cfDNA concentration of cells treated with 10%, 2.5%, 0% FBS. Our results suggested that cfDNA concentration had a positive relation with the percent of cells in G1 phase.

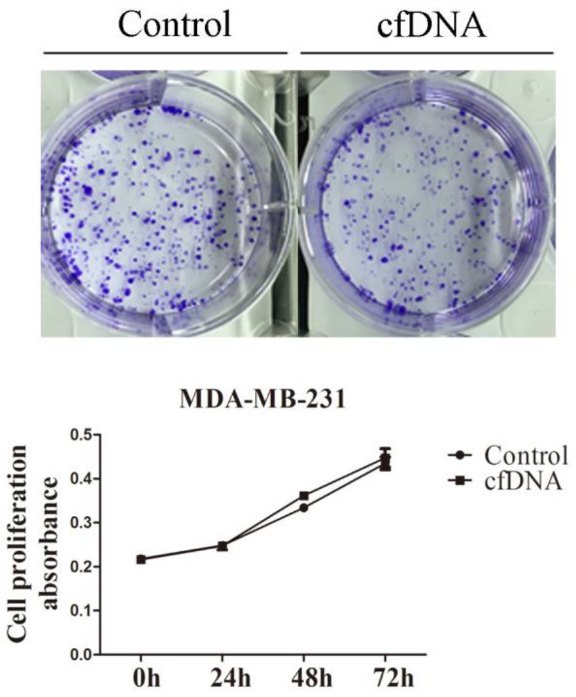


**Supplementary Figure 3: Proliferation promotion effect of cfDNA on MDA-MB-231 cells.** Clone formation assay and CCK-8 assay showed cfDNA could not promote the proliferation in MDA-MB-231 cells.

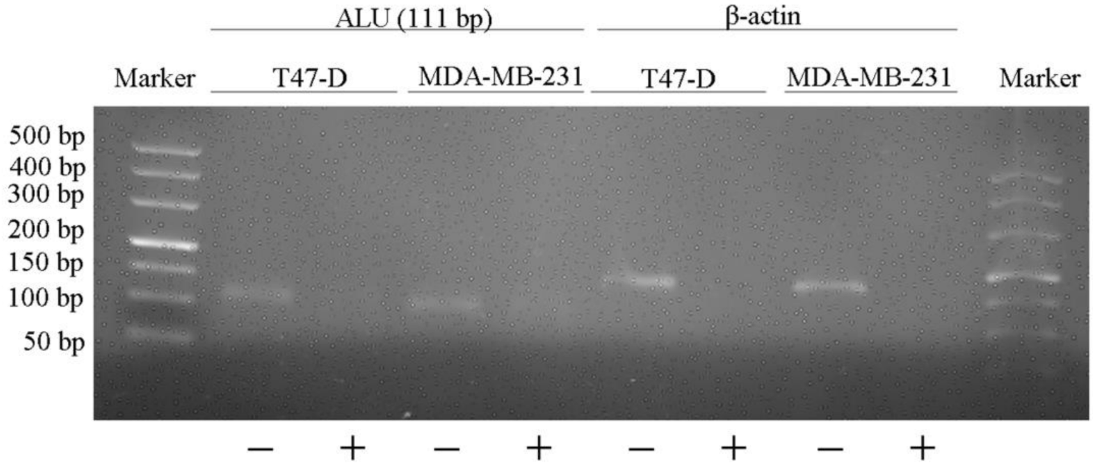


**Supplementary Figure 4: An agarose gel electrophoresis has been done to prove that DNA is digested by DNase I indeed.** In this experiment, DNase I was added into the supernatant of T47-D and MDA-MB-231 cell. Then cfDNA was extracted. ALU gene and β-actin gene were amplified with PCR. Agarose gel electrophoresis was used to detected the amount of genes. The result showed that DNase Icould digest DNA sufficiently. +: supernatant processed with DNase I, –: supernatant not processed with DNase I.

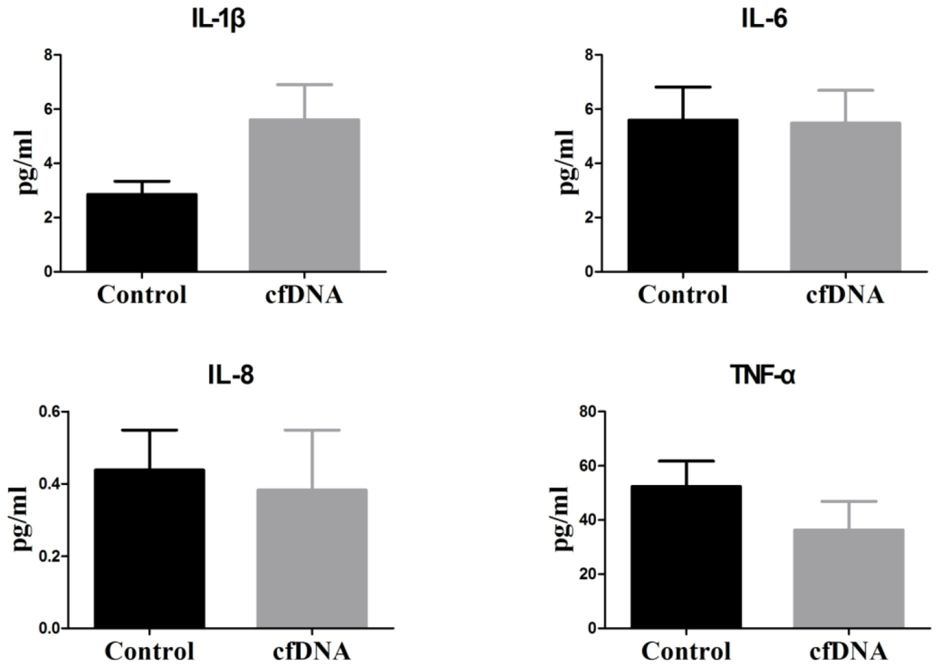


**Supplementary Figure 5: The levels of inflammatory cytokines in the supernatant of T47-D cells.** We assessed the levels of IL-1β, IL-6, IL-8 and TNF-α, which can be activated through NF-κB pathway. Our results showed that the level of IL-1β increased after cells were treated with cfDNA, but had no statistical significance. The IL-6 level of the cfDNA group had no difference with the control group. IL-8 and TNF-α even decreased in the cfDNA group but had no statistical significance.
